# Additive and Laser Manufacturing for Multifunctional Electronics on High‐Performance Polymers

**DOI:** 10.1002/smsc.202500022

**Published:** 2025-04-02

**Authors:** Joshua Vandervelde, Yeowon Yoon, Rifat Shahriar, Stephen B. Cronin, Yong Chen

**Affiliations:** ^1^ Center for Advanced Manufacturing University of Southern California Los Angeles CA 90007 USA; ^2^ Department of Aerospace and Mechanical Engineering University of Southern California Los Angeles CA 90089 USA; ^3^ Department of Electrical Engineering University of Southern California Los Angeles CA 90089 USA; ^4^ Department of Physics and Astronomy University of Southern California Los Angeles CA 90089 USA; ^5^ Department of Chemistry University of Southern California Los Angeles CA 90089 USA; ^6^ Department of Industrial and Systems Engineering University of Southern California Los Angeles CA 90089 USA; ^7^ Department of Biomedical Engineering University of Southern California Los Angeles CA 90089 USA

**Keywords:** additive manufacturings, heaters, laser‐induced graphenes, polyether ether ketones, polyetherimides, strain sensors

## Abstract

Laser‐induced graphene (LIG) is a novel multifunctional material fabricated from a single‐step laser scribing process on a variety of polymers. LIG electronics display exceptional conducting, heating, and sensing properties, which are desirable for customizable circuits within 3D‐printed structures. However, the properties of LIG on high‐performance additive manufacturing (AM) materials, such as polyetherimide (PEI, trade name Ultem) and polyether ether ketone (PEEK), have not been thoroughly investigated. In this study, LIG is scribed by a blue laser on pure and 3D‐printed PEI and PEEK. Remarkably, the LIG's electrical performances represent several of the lowest sheet resistances reported on PEI‐ and PEEK‐derived LIG to date. These minimal values (1.02 Ω sq^−1^) and their high conductivities (45.4 S cm^−1^) are also among the best electrical characteristics studied on any LIG precursor. The versatility of LIG electronics for AM is further demonstrated on 3D‐printed specimens with laser‐scribed heaters and strain gauges. LIG heaters show impressive operating ranges and excellent electrothermal properties; LIG strain gauges exhibit large gauge factors and minimal drift. In these findings, an effective approach to fabricate facile electronics in AM structures by integrating additive and laser manufacturing processes is presented.

## Graphical abstract

Highly conductive laser‐induced graphene (LIG) is scribed by a low‐power blue laser on additively manufactured (AM) polyetherimide and polyether ether ketone. Functional and sensing LIG electronics are investigated through Joule heaters and strain gauges, which operate reliably under high thermal and mechanical stresses. In these findings, the embedding of facile electronics in high‐performance AM structures is motivated.

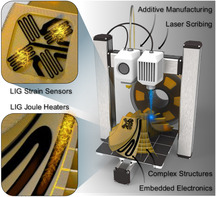

## 1 Introduction

Since its discovery in 2014,^[^ ^1^ ^]^ laser‐induced graphene (LIG) has demonstrated remarkable thermal, electrical, mechanical, and chemical properties for applications involving flexible heaters,^[^ ^2^, ^3^, ^4^ ^]^ wearable electronics,^[^ ^5^, ^6^, ^7^ ^]^ supercapacitors,^[^ ^8^, ^9^, ^10^ ^]^ physical sensors,^[^ ^11^, ^12^, ^13^ ^]^ and biochemical sensors.^[^ ^14^, ^15^, ^16^ ^]^ Fabricated using a one‐step laser‐scribing process in ambient conditions, LIG has the potential to significantly simplify electronics manufacturing on multifunctional surfaces.^[^ ^17^ ^]^ When a laser ablates a precursor surface that contains aromatic compounds, porous, multilayer graphene is formed due to the molecular lattice vibration of the substrate with the laser. A rapid increase in thermal energy^[^ ^18^ ^]^ breaks the precursor's C—O, C=O, and C—N bonds to release gaseous CO, H_2_, and C_x_H_y_N_z_ hydrocarbons,^[^ ^1^, ^19^ ^]^ resulting in a graphitic rearrangement of *sp* ^2^‐hybridized carbon. Moreover, from the sudden expulsion of gas, the enlargement of graphene flakes by residual heat,^[^ ^18^ ^]^ and the contortion of 5‐ and 7‐member rings in the otherwise hexagonal carbon lattice, the graphene induced by this process usually forms a foamy 3D structure.^[^ ^20^ ^]^ This unique surface morphology, combined with the graphene's high electrical and thermal conductivities,^[^ ^2^, ^3^, ^4^, ^5^, ^6^, ^7^ ^]^ gives LIG excellent sensing and energy storage capabilities with rapid response rates.^[^ ^8^, ^9^, ^10^, ^11^, ^12^, ^13^, ^14^, ^15^, ^16^ ^]^

LIG was originally ablated using infrared (IR) CO_2_ lasers (*λ* = 10.6 μm) at powers exceeding 2 W and often upward of 10 W.^[^ ^1^, ^21^ ^]^ Recent research has investigated the effects of creating LIG with low‐power and short‐wavelength lasers that are readily accessible to the public at a cost‐effective price. Visible spectrum lasers, notably the blue laser (*λ* = 400–500 nm),^[^ ^22^, ^23^ ^]^ have been shown to induce LIG with powers as low as 0.1 W.^[^ ^12^ ^]^ This dramatic decrease in power owes primarily to the smaller spot sizes of shorter wavelength lasers. Conducted across a variety of substrates, LIG scribed by blue lasers has been reported with similar electrical, structural, and functional characteristics to LIG scribed by IR lasers^[^ ^5^, ^21^, ^24^ ^]^ while having spatial resolutions up to five times smaller.^[^ ^23^ ^]^ Blue lasers’ low power requirements and small diode sources also make equipment for LIG manufacturing more compact. Recently, blue laser diodes have been integrated into desktop positioners with five degrees of freedom for conformal LIG scribing,^[^ ^25^, ^26^ ^]^ a task that would be challenging for larger laser sources.

Despite these advances, few studies have investigated LIG scribing on additive manufacturing (AM) materials with complementary properties to LIG, particularly polyetherimide (PEI) and polyether ether ketone (PEEK).^[^ ^27^, ^28^, ^29^ ^]^ These high‐performance polymers are widely used in engineering applications: both thermoplastics boast high elastic moduli, tensile strengths, chemical resistances, and thermal stabilities.^[^ ^30^, ^31^ ^]^ These materials and their scribed LIG patterns can consequentially withstand and measure high stresses, tolerate and detect strong chemicals, and endure and produce extreme temperatures. A previous study using dynamic molecular simulations^[^ ^19^ ^]^ has also shown that LIG derived from PEI and PEEK possesses the highest internal surface area per volume compared to LIG from other commercial polymers, enhancing its sensing and energy‐transfer capabilities. Moreover, components with high geometric complexity from both thermoplastics can be easily formed using fused deposition modeling (FDM) 3D printing, allowing functional LIG patterns to be integrated into 3D‐printed structures. Commercially available PEI and PEEK filaments for FDM printing are already emerging in final product manufacturing, spurring certifications of the materials in food, medical, and aerospace industries by the National Sanitation Foundation (NSF), Food and Drug Administration (FDA), and Federal Aviation Administration (FAA).^[^ ^32^, ^33^, ^34^ ^]^

LIG has been reported on pure and 3D‐printed PEI^[^ ^27^, ^35^ ^]^ and PEEK^[^ ^28^, ^36^ ^]^ using green (*λ* = 532 nm) and IR laser systems, but to the best of the authors’ knowledge, the synthesis of LIG from these materials using a blue laser has never been reported. Customizable structures with integrated sensors or tunable features, like heating^[^ ^2^ ^]^ and water repelling,^[^ ^17^ ^]^ have long been sought by AM researchers. However, the AM field has been limited by multi‐material approaches requiring complex filament, resin, or powder exchanges.^[^ ^37^, ^38^ ^]^ Attempts to incorporate LIG in AM have faced similar challenges: multi‐step manufacturing processes are required for direct ink writing using dissolved LIG flakes,^[^ ^39^ ^]^ powder bed fusion using sintered substrates,^[^ ^40^ ^]^ and sheet lamination using multiple lasers.^[^ ^41^ ^]^ By integrating 3D printing and laser scribing, structural and functional material can instead be fabricated in tandem without material or tool changes. Still, the unification of LIG scribing and 3D printing has been challenged by poor LIG conductivity^[^ ^25^ ^]^ while improvements to electrical performance have been offset by the complexity of postprocessing steps^[^ ^26^ ^]^ and the sizing and power requirements of IR laser systems.^[^ ^35^, ^36^, ^42^, ^43^ ^]^

In this study, high‐quality LIG is shown, for the first time, to be scribed on pure and 3D‐printed PEI and PEEK polymers using a 450 nm laser. This blue laser is an unlikely candidate for these materials, especially while creating LIG with laser fluences as low as 12 J cm^−2^: both PEI and PEEK exhibit minimal absorbances around *λ* = 450 nm.^[^ ^44^, ^45^ ^]^ By contrast, prevailing theories on the photothermal formation of LIG suggest that lower precursor absorbances require higher critical fluences for LIG to appear.^[^ ^5^ ^]^ However, polyimide strongly absorbs blue light and still requires a critical fluence of more than 80 J cm^−2^ for the LIG growth at this wavelength.^[^ ^23^ ^]^

**Figure 1:**
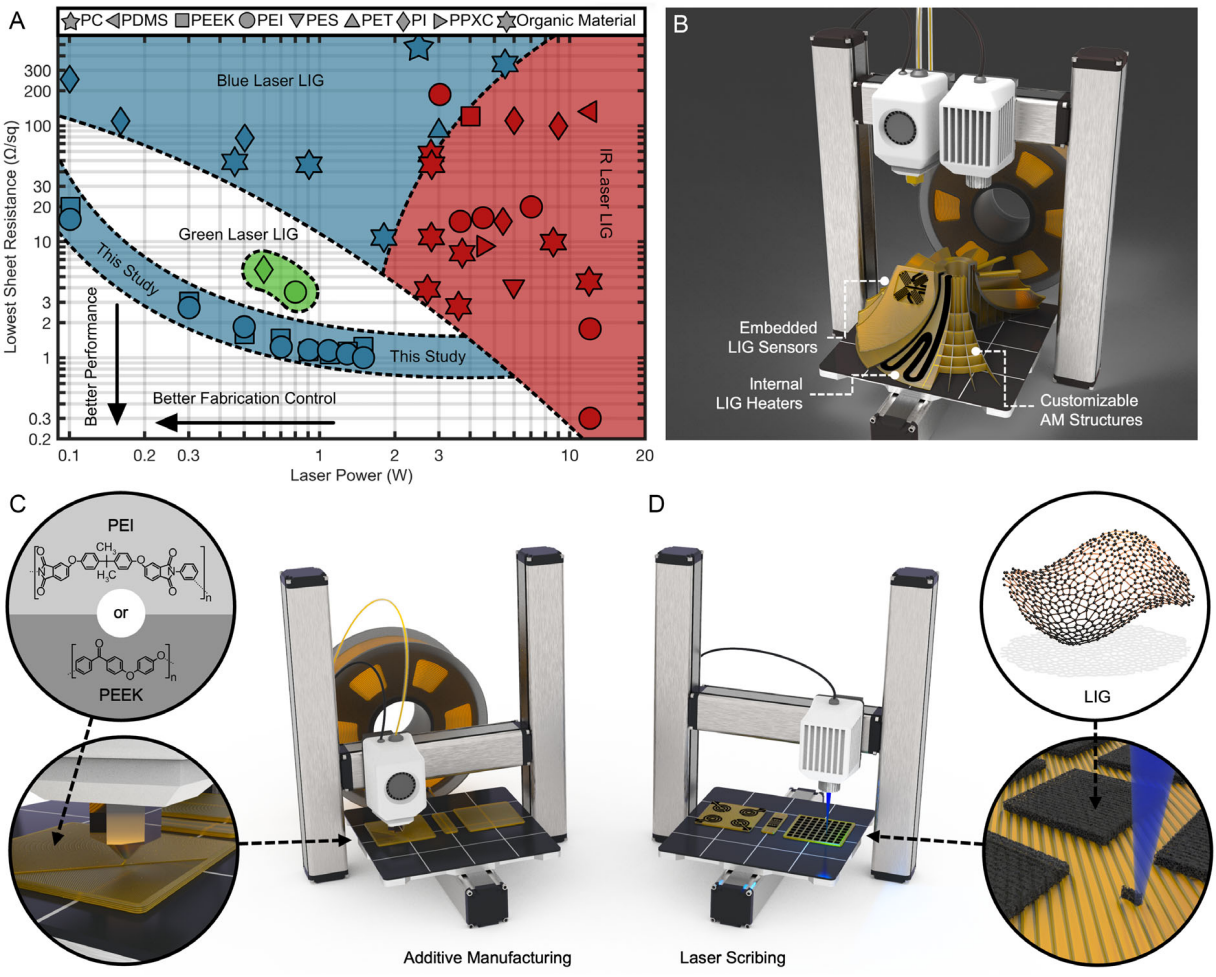
A) Comparison of LIG sheet resistances, laser powers, laser wavelengths, and precursor materials used in other studies against those of this work. Standard abbreviations of polymer names are used. B) Envisioned dual additive and laser manufacturing process for embedded LIG electronics in customizable structures; cross sections of a 3D‐printed impeller pump reveal internal LIG circuits. C) Representation of the methods used to create 3D‐printed PEI and PEEK samples with D) LIG electronics scribed by a blue laser; arrows guide between the molecular‐, micro‐, and macroscale structures of each fabrication process.

Furthermore, this study achieves conductivities as high as 45.4 S cm^−1^ from LIG on pure PEI, pure PEEK, 3D‐printed PEEK, 3D‐printed Ultem 1010 (derived from PEI), and 3D‐printed Ultem 9085 (derived from PEI). These findings represent the lowest sheet resistances reported on LIG from four of five examined materials, with values dropping to 1.02 Ω sq^−1^ (Table S1, Supporting Information). In addition, the minimal sheet resistances of LIG from PEI and PEEK at eight laser powers (0.1–1.5 W) were among the lowest values reported on any LIG precursor using any laser power (**Figure** **1**A). These findings overcome a previous trade‐off between LIG sheet resistance and formative laser power by achieving desirable electrical properties and beneficial manufacturing merits. Reduced sheet resistances improve energy efficiency, sensor response, and signal integrity for LIG electronics, while reduced laser powers enhance spatial precision, fluence control, and human safety during fabrication.

Future AM systems with integrated laser systems (Figure 1B) will also benefit from these consistent and stable (±0.20 Ω sq^−1^) sheet resistances of LIG over a breadth of laser powers (0.5–1.5 W). To embed LIG electronics in 3D‐printed PEI and PEEK, a wide range of permissible powers will allow integrated lasers to be chosen based more on beam precision, equipment size, and thermal tolerance. To demonstrate the multifunctionality of LIG electronics in AM structures, electric heaters and strain gauges were laser‐scribed on 3D‐printed PEEK, Ultem 1010, and Ultem 9085 surfaces. These applications exhibited high operating limits and consistent, predictable responses that were complementary to the robust thermal and mechanical properties of their substrates. These findings aim to inspire further research into seamlessly embedded electronics in 3D‐printed structures using high‐performance polymers via additive and laser manufacturing.

## 2 Results and Discussion

### 2.1 Synthesis of Materials

High‐temperature FDM printers were chosen to fabricate the 3D‐printed PEI and PEEK, as the polymers’ superior melting and glass transition temperatures restricted the selection of nozzles, build plates, and heated chambers that could handle them. Ultem 1010 and Ultem 9085 filaments were extruded by an F900 printer (Stratasys, Eden Prairie, MN), while the PEEK filament was printed by a 22 IDEX printer (Vision Miner, Santa Ana, CA). Despite the ease by which these 3D printers could achieve complex geometries, all samples were fabricated in rectangular patterns with a uniform thickness of 1.59 mm (Figure 1C). These choices allowed the pure, commercially procured PEI and PEEK sheets to be cut and ground to the same dimensions. The fixed thickness of all samples also allowed the LIG–polymer interface to be observed, which may have otherwise been obscured by additional topologies.

Each pure and 3D‐printed sample was then affixed to a 3‐axis positioning system outfitted with a blue diode laser (*λ* = 450 nm). Once optically focused, this pulsed laser ablated the polymer surface according to a programmed path to induce LIG (Figure 1D). Laser parameters were chosen according to a preliminary analysis of these materials: the laser power *P*_L_ and its pulse duration *τ*_L_ were lowered on each polymer until LIG did not form, which occurred near either *P*_L_ = 0.1 W or *τ*_L _= 1 ms or both. For the rest of this study, powers between 0.1 and 1.5 W were used with pulse durations between 1 and 8 ms.

Other parameters, such as the laser's spot size, spacing between spots, and laser raster pattern, were kept consistent between samples. The blue laser had a 1*e* ^−2^ radius of *w* = 100 μm; however, the radii of LIG spots were observed at a reduced ≈50 μm. This difference was likely owed to the Gaussian profile of the laser intensity, where the beam center had a higher fluence to induce LIG photothermally. The center‐to‐center distance between adjacent LIG spots was set to 50 μm so that they overlapped, emulating the effect of scribing LIG patterns with multiple passes. Consequentially, every LIG pattern was scribed an equivalent of *n* = 3.14 times at this distance. This corresponded to previous studies on PEI and PEEK, which found that 3–5 laser passes on these materials produced the highest‐quality LIG.^[^ ^36^, ^46^ ^]^ These laser parameters allowed the laser's peak fluence, Φ_*0*_, to be calculated by Equation (1).

(1) $$\Phi_{0}=\frac{P_{L}\tau_{L}n}{\pi w^{2}/2}$$

### 2.2 Material Characterization

**Figure 2:**
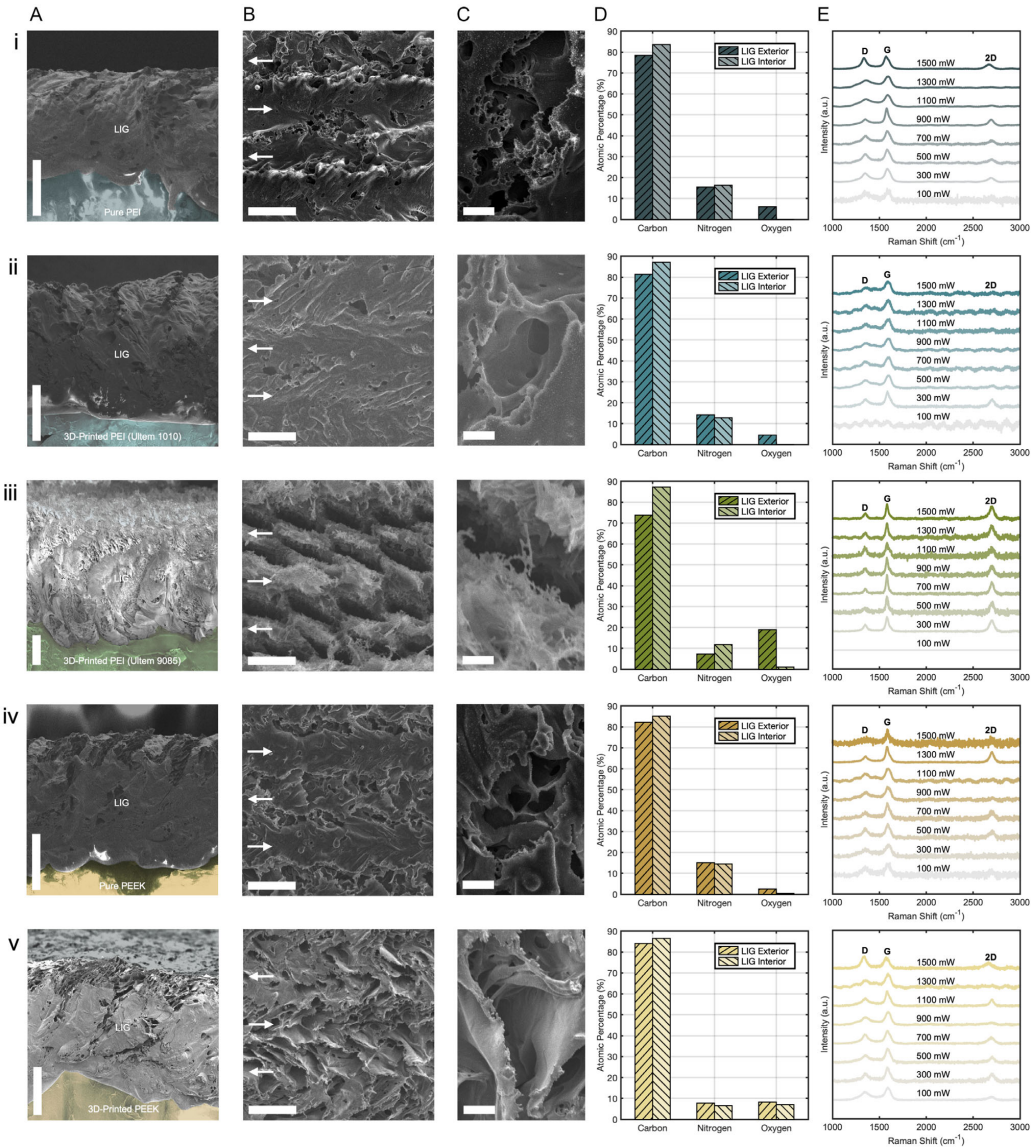
A) Side‐view SEM images of LIG–polymer interfaces with false coloring; scale bars, 100 μm. B) Top‐view SEM images of LIG; scale bars, 50 μm. Arrows indicate the centers and directions of adjacent laser passes during fabrication. C) Magnified top‐view SEM images of crevices on the LIG surfaces; scale bars, 10 μm. D) EDS averages of atomic compositions for the LIG exterior and interior. E) Raman spectra of LIG scribed by different laser powers. Measurements were performed on the same LIG precursor for each row: i) pure PEI; ii) 3D‐printed Ultem 1010; iii) 3D‐printed Ultem 9085; iv) pure PEEK; and v) 3D‐printed PEEK.

Scanning electron microscopy (SEM) images of LIG samples were taken from top and side views to determine their surface morphologies and thicknesses. Each of the five precursor materials was cut into 5 by 5 mm squares, ablated with the laser's maximum fluence (Φ_0 _= 240 J cm^−2^ at *P*_L_ = 1.5 W and *τ*_L _= 8 ms), and scraped with a razor to reveal clean interfaces between the LIG and polymer. False‐color SEM cross sections of these interfaces showed the LIG structures, dense with interwoven sheets and speckled by isotropic pores, nested on top of PEI and PEEK substrates (**Figure** **2**A). The formation of these stratified LIG layers was unusual; SEM profiles of LIG are typically dominated by their highly porous structures and lack of smooth surfaces.^[^ ^5^, ^20^ ^]^ Growth patterns that have displayed fewer external pores are often attributed to insufficient laser fluences and high gas retention (oxygen concentrations ≥ 30%).^[^ ^20^, ^47^ ^]^ However, all LIG samples (Figure 2A–C) were ablated with a laser fluence 20 times that of their critical fluence and demonstrated low interior oxygen concentrations (≤8.2%) (Figure 2D). To explain these morphologies, previous studies using reaction force field simulations have shown LIG from PEI and PEEK to form a higher concentration of 6‐member carbon rings than other polymers.^[^ ^19^, ^48^ ^]^ Low defect concentrations in the graphene's atomic structure, which 5‐ and 7‐member rings inhibit, promote planar crystalline growths and may give rise to these LIG sheets.

Swaths of unbroken LIG appeared most prominently on the top surfaces of pure PEI, 3D‐printed Ultem 1010, and pure PEEK (Figure 2B). Considering the alternating direction of ridges between flat strips and their ≈50 μm offsets, LIG sheets were likely formed in the center of the laser's path as it crisscrossed the underlying substrate. On 3D‐printed PEEK, large sheets of LIG also appeared but were layered out of the plane perpendicular to the laser beam's direction. From higher‐magnification SEM images (Figure 2C), sheets with thicknesses ranging from tenths of a micrometer to single‐digit micrometers were seen. These denser structures likely facilitated superior electrical properties over porous LIG: continuous electrical pathways reduced sheet resistance and lowered the defect scattering of charge carriers to increase LIG conductivity.

The top surface of the LIG on 3D‐printed Ultem 9085 did not display this sheetlike behavior but instead developed a sparsely reticulated network of LIG fibers (Figure 2B). Though the side profile of this structure exhibited deeper, denser formations, the top ≈70 μm only created weblike LIG in the center of each laser pulse. It also spread out more than growths on other polymers; the average thickness *d* of LIG on Ultem 9085 was 458 μm, compared to 287 μm on 3D‐printed PEEK, 270 μm on Ultem 1010, 235 μm on pure PEEK, and 219 μm on pure PEI (Figure S1, Supporting Information). This morphological difference may have been influenced by the high concentration of polycarbonate in Ultem 9085, which allows it to be 3D printed at a lower temperature and have higher flowability^[^ ^49^ ^]^ than other PEI derivatives like Ultem 1010. Polycarbonate converts poorly to LIG when scribed by a laser,^[^ ^26^, ^46^ ^]^ so the additional degassing of its liberated atoms may have increased the expansion of Ultem 9085's LIG as it emerged.

Multiple energy‐dispersive spectroscopy (EDS) measurements were taken on and under the surface of each LIG sample to determine their atomic compositions (Figure 2D). Averages over multiple locations showed a clear trend of carbon concentrations increasing toward the LIG interior, likely spurred by the decrements in oxygen. While carbonization generally increases with laser fluence,^[^ ^20^ ^]^ the lack of oxygen in several LIG interiors and the highest cumulative fluence at the LIG surface indicated exterior oxidation as the strongest candidate for this trend.^[^ ^1^ ^]^ The high oxidation on the LIG exterior of Ultem 9085 is likely due to the increased surface area of its fiber network, allowing for more covalent C—O bonds to form per volume.^[^ ^50^ ^]^ By the same reasoning, 3D‐printed PEEK showed less exterior oxidation per volume from its angled sheet morphology, followed by minimal oxidations on the flat exteriors of pure PEEK, pure PEI, and Ultem 1010.

Though nitrogen stayed relatively constant between the LIG's surface and bulk, its atomic concentration in all samples was markedly high. Average nitrogen concentrations of LIG scribed by the blue laser reached past 16.3% on PEI substrates and 15.1% on PEEK (Figure 2D); these concentrations in LIG from green and IR lasers have only been shown up to 5.8% for PEI^[^ ^27^, ^48^ ^]^ and not past 0% for PEEK.^[^ ^28^, ^36^ ^]^ Moreover, the nitrogen content of blue‐laser LIG far exceeded the original content in its precursors’ chemical structures: 2.9% for PEI and 0% for PEEK (Figure 1C). This implies that ambient N_2_ molecules were ionized during the extreme exothermic formation of LIG and formed covalent C—N bonds with the carbon lattice to produce pyridinic, pyrrolic, and quaternary nitrogen inclusions. Since the higher photon energies of shorter laser wavelengths have been shown to increase nitrogen concentrations in LIG from other polymers,^[^ ^16^ ^]^ the blue laser used in this study may be solely responsible for the large nitrogen influx in LIG from PEI and PEEK. The increased carrier concentration and free electron mobility in the nitrogen‐doped graphene^[^ ^51^ ^]^ played key roles in the LIG's excellent conductivity and minimal sheet resistance.

Raman spectroscopy was performed on LIG samples scribed by eight laser powers (*P*_L_ = 0.1–1.5 W, *τ*_L _= 8 ms) on all five precursor polymers (Figure 2E). Characteristic peaks in the spectra signals, conducted over wavenumbers from 1000 to 3000 cm^−1^, were used to identify graphene compositions in these samples and analyze their resonance modes. Prominent peaks in the D band at ≈1350 cm^−1^, the G band at ≈1580 cm^−1^, and the 2D band at ≈2700 cm^−1^ indicated the presence of graphene defects, *sp* ^2^‐hybridized carbon, and electronic band features, respectively.

The intensity ratios between the 2D and G bands were calculated to compare the extent of multilayered graphene and electron doping in each LIG sample (Figure S2, Supporting Information). When graphitic layers are added to monolayer graphene, the increased coupling of interlayer electronic bands dampens the intensity of pure 2D band vibrations and reduces the *I*_2D_/*I*_G_ ratio. As a result, the *I*_2D_/*I*_G_ ratios from Ultem 9085's LIG showed the highest average values of ≈0.72, followed by those of pure PEEK, 3D‐printed PEEK, Ultem 1010, and pure PEI. This sequence logically matched the descending order of LIG's morphological complexity: Ultem 9085 exhibited the most porous LIG structure, agreeing with *I*_2D_/*I*_G_ inferences of reduced graphene layers. Conversely, pure PEI and Ultem 1010 produced denser LIG structures, which agreed with their low *I*_2D_/*I*_G_ ratios and thus elevated graphene stacking. However, heightened electron concentrations of nitrogen‐doped graphene also lessened *I*_2D_/*I*_G_ ratios due to increased Fermi levels and Pauli blocking.^[^ ^51^ ^]^ The minimal *I*_2D_/*I*_G_ ratios of LIG from pure PEI (≈0.06), Ultem 1010 (≈0.17), 3D‐printed PEEK (≈0.18), and pure PEEK (≈0.21) were all likely diminished by this nitrogen doping and electron saturation.

The pattern relating the *I*_D_/*I*_G_ ratios of LIG samples to their laser fluences was less distinct. Lower intensity ratios between Raman spectra's D and G peaks indicate a higher graphene quality from fewer atomic defects. This value directly corresponds to the average length of crystalline growths, *L*_a_ (nm), along their *a* axis by the Tuinstra–Koenig relation in Equation (2). The wavelength of the excitation laser used for Raman spectroscopy was *λ*_L_ = 532 nm.

(2) $$L_{a}=\left(2.4\;\times \;10^{-10}\right)\;\lambda_{L}^{4}{\left(\frac{I_{D}}{I_{G}}\right)}^{-1}$$

The *I*_D_/*I*_G_ ratio generally decreased in the central 0.3–1.3 W power range, promoting graphene growths with larger crystalline lengths (Figure S2, Supporting Information). Powers lower and higher than these produced increase in the *I*_D_/*I*_G_ ratio for nearly all materials. This trend has been shown on LIG across a variety of other precursor materials, owing to insufficient fluences or energies high enough to damage the LIG.^[^ ^4^, ^18^, ^28^ ^]^ The defect concentrations of shortened *L*_a_ distances also corresponded to the intensity of nitrogen doping in each LIG sample, with reduced inter‐defect lengths occurring on the most heavily doped graphene. However, static Raman shifts showed low correlations between the *I*_D_/*I*_G_ ratios of LIG and their formative laser fluences on linear (*R* ^2 ^≈ 0.08) and polynomial (*R* ^2 ^≈ 0.28) best‐fit curves (Figure S3, Supporting Information). Either laser fluence had a reduced influence on the graphene quality of LIG, or graphitic properties within a single LIG sample varied considerably.

### 2.3 Electrical Properties

**Figure 3:**
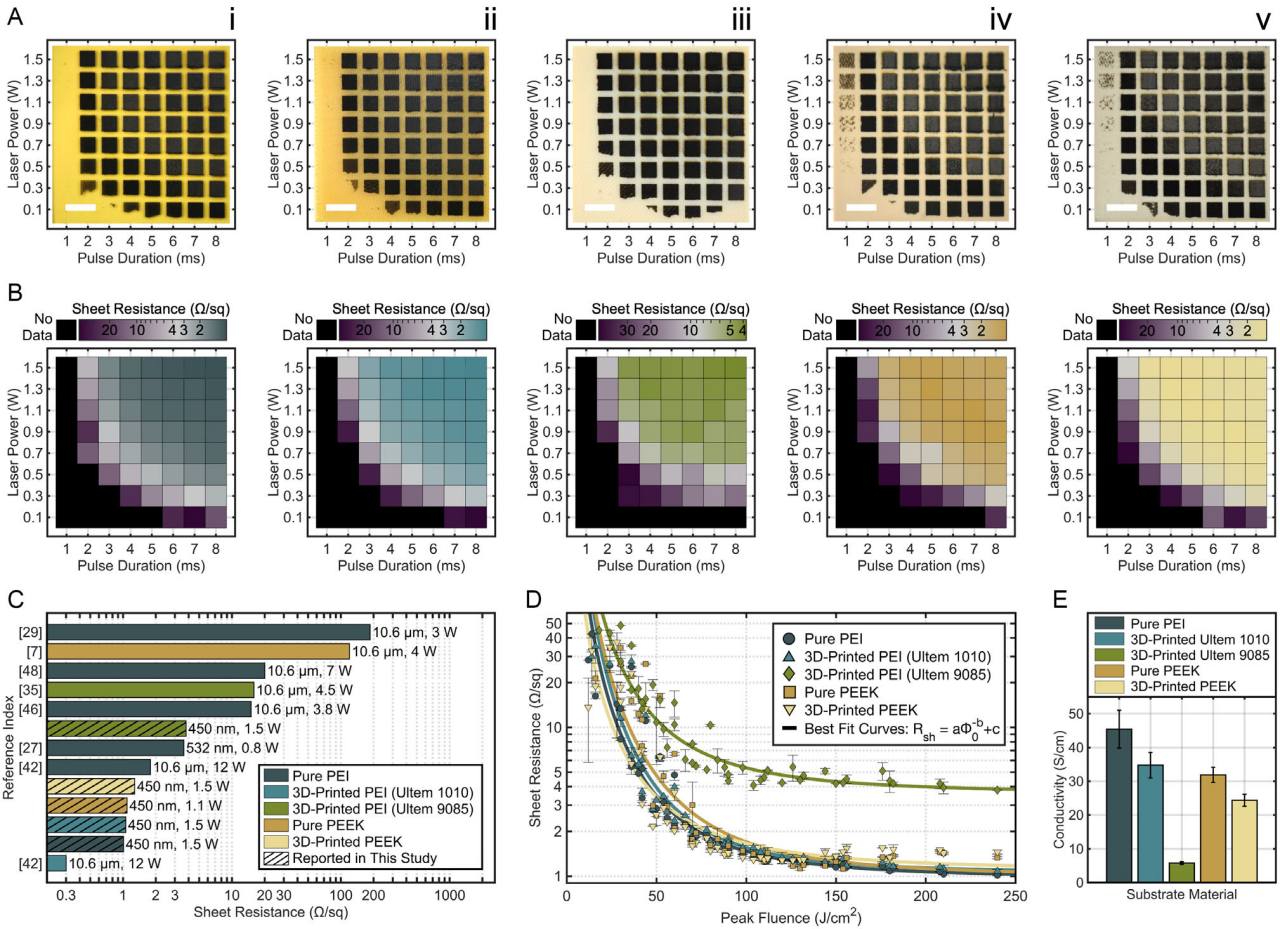
A) LIG scribed by different combinations of laser power and pulse duration on each polymer; scale bars, 10 mm. All LIG precursors are ordered as i) pure PEI; ii) 3D‐printed Ultem 1010; iii) 3D‐printed Ultem 9085; iv) pure PEEK; and v) 3D‐printed PEEK. B) Heatmaps of average sheet resistances on each LIG sample; samples without data had large and/or highly variable sheet resistances. C) Comparison of lowest LIG sheet resistances, laser wavelengths, and laser powers reported in this work against other studies on PEI and PEEK. D) Sheet resistance is a function of peak laser fluence; mathematical power functions best fit each precursor's data. E) The conductivity of LIG was calculated for each precursor at the maximum laser fluence examined.

Sheet resistance, *R*_sh_, is an important electrical characteristic that measures the bulk resistivity of a material per thickness. For a thin conductor with a set thickness, such as LIG, reduced sheet resistances benefit electrical circuits and can be easily compared to other reported values. These qualities appeal to applications requiring high energy efficiency, low voltage, or strong signal transmission; even small decrements in sheet resistance can drastically improve performance. To understand the effects of laser power and pulse duration on LIG sheet resistance, a combination matrix of laser powers (*P*_L_ = 0.1–1.5 W) and pulse durations (*τ*_L _= 1–8 ms) was used to scribe each of the five substrate polymers (**Figure** **3**A). PEEK and PEI are among the best electrical insulators of all polymers (10^16^–10^18^ Ω cm), so the electrical influence between LIG patterns was negligible. Sheet resistances were measured twice on each LIG sample with a four‐point probe and averaged for plotted data.

Across all materials, the gridded specimens lacked LIG along their bottom and left edges, where laser fluence was the lowest. Several regions showed partial conversion to LIG, associated with near‐critical fluences, causing LIG nucleation sites to stochastically emerge.^[^ ^47^ ^]^ In some cases, larger nucleation seeds promoted subsequent and adjacent LIG growths, as seen with incomplete LIG squares on the *P*_L_ = 0.1 W row of all the polymer substrates. In other cases, nucleation sites formed sporadically but did not fully merge, as seen with dotted LIG patterns along the *τ*_L_ = 1 ms column of both PEEK precursors. However, sheet resistance was uniformly reduced with increased laser power and pulse duration among laser parameters that yielded coherent LIG squares. This trend is visually apparent on heatmaps of logarithmic sheet resistance (Figure 3B) underneath each corresponding material image (Figure 3A): the top right quadrant of every heatmap quickly stabilized around a representative color.

Remarkably, most of these optimal LIG sheet resistances represent the lowest values reported on each material to date. With a minimal 1.02 Ω sq^−1^ on pure PEI, 1.12 Ω sq^−1^ on pure PEEK, 1.28 Ω sq^−1^ on 3D‐printed PEEK, and 3.79 Ω sq^−1^ on 3D‐printed Ultem 9085, these values drop below previous records (Figure 3C) by wide margins (107 times smaller on PEEK; 1.75 times smaller on PEI). LIG scribed on 3D‐printed Ultem 1010 achieved the second lowest sheet resistance of its material, at a value of 1.08 Ω sq^−1^. Favorably, the powers used to create LIG by this blue laser were much lower than those used by IR lasers.

The minimum sheet resistances found in this study were among the lowest reported values for LIG from any precursor material^[^ ^5^, ^52^ ^]^ (Table S1, Supporting Information). However, these qualities were not limited to finely tuned synthesis parameters. For more than half of all measured samples, LIG sheet resistances were less than 1.56 Ω sq^−1^ on 3D‐printed PEEK, 1.70 Ω sq^−1^ on pure PEI, 1.78 Ω sq^−1^ on pure PEEK, 1.79 Ω sq^−1^ on Ultem 1010, and 6.07 Ω sq^−1^ on Ultem 9085. These values signify a broad range of laser powers and pulse durations to choose from while still achieving excellent electrical properties.

To predict the sheet resistance of LIG as a function of its laser fluence, mathematical power functions (Equation (3)) were best fit to each material's data (Figure 3D). The experimental data exhibited a strong exponential decay with physically significant asymptotes on both positive axes. However, no theory has yet derived a comprehensive equation between laser fluence and LIG sheet resistance, leaving empirical results to make best‐fit models. Previous reports have exhibited similar nonlinear curves between these terms,^[^ ^53^ ^]^ and one recent study suggested a two‐coefficient power function to represent their trends.^[^ ^27^ ^]^ The model presented here takes a step further by introducing a constant offset coefficient, signifying that LIG cannot have sheet resistances lower than a threshold value specific to each material and process parameter.

(3) $$R_{\text{sh}}=a\Phi_{0}^{-b}+c$$

This three‐term model closely matched sheet resistance measurements, with correlations as strong as *R* ^2^–0.94. The lowest threshold coefficients *c* for pure PEI and PEEK indicated that they would achieve the lowest asymptotic sheet resistances, followed by Ultem 1010, 3D‐printed PEEK, and finally Ultem 9085 (Figure S4, Supporting Information). This order differed from experimental values, which may have been influenced by statistical deviations. The model's decay rates also proved insightful: the highest rate, *b* = 2.50 on Ultem 1010's LIG, suggested that its sheet resistance would diminish ≈5.7 times relative to its threshold value for every doubling in laser fluence.

Coefficients and *R* ^2^ values for this model were calculated from the linearized logarithmic form of Equation (3) to weigh sheet resistances equally against each other, preventing larger fluctuating values from dominating their least squares regressions. Despite high correlations to experimental data, this empirical model does not account for the degradation of LIG quality at higher laser fluences. Past ≈130 J cm^−2^, both PEEK‐derived polymers exhibited a slight increase in sheet resistance before stabilizing over the next ≈100 J cm^−2^. If laser fluence were to be increased beyond the scope of this study, the atomic quality of *sp* ^2^ carbon would diminish,^[^ ^20^ ^]^ resulting in much higher sheet resistances.^[^ ^28^ ^]^

LIG conductivity was calculated at the highest fluence conditions (Φ_0 _= 240 J cm^−2^ at *P*_L_ = 1.5 W and *τ*_L _= 8 ms), which were used to create all LIG samples for the remainder of this study. Although minimum sheet resistances were found at other fluences for both PEEK polymers, this fluence was chosen to easily compare the five precursor materials. Conductivity is directly related to sheet resistance *R*_sh_ and material thickness *d* (Equation (4)), so trends seen in prior analyses are shared here.

(4) $$\sigma =\frac{1}{R_{\text{sh}}d}$$

LIG on pure PEI demonstrated the highest conductivity at 45.4 S cm^−1^, followed by Ultem 1010 at 34.8 S cm^−1^, pure PEEK at 31.9 S cm^−1^, 3D‐printed PEEK at 24.4 S cm^−1^, and Ultem 9085 at 5.79 S cm^−1^ (Figure 3E). The large difference in conductivity on Ultem 9085's LIG owed partially to its surface morphology, where its porous, fibrous structure impeded electron flow and caused carrier scattering. However, changes in conductivity were more strongly correlated to the nitrogen concentrations of each precursor's LIG, along with the enhanced electron and atomic defect concentrations attributed to nitrogen doping (Figure S5, Supporting Information). Across all specimens, conductivity increased linearly with nitrogen concentrations (*R* ^2 ^≈ 0.74), causing both the carbon (*R* ^2 ^≈ 0.54) and oxygen (*R* ^2 ^≈ 0.72) contents to proportionally decrease. In turn, elevated conductivity of the LIG's molecular lattice decreased the Raman spectra *I*_2D_/*I*_G_ ratio (*R* ^2 ^≈ 0.90) from the 2D band's muted resonance due to increased electron concentrations.^[^ ^51^ ^]^ The Raman *I*_D_/*I*_G_ ratio was equivalently heightened with rising conductivity (*R* ^2 ^≈ 0.73) from higher concentrations of nitrogen defects in the graphene structure. Since both intensity ratios were directly and proportionally influenced by nitrogen doping, a composite *I*_D_/*I*_G_ + *I*_2D_/*I*_G_ ratio was compared to LIG conductivity with a markedly high correlation (*R* ^2^ > 0.99). This previously unexplored composite ratio may be beneficial to characterize future LIG whose enhanced conductivity is directly influenced by high nitrogen doping concentrations.

### 2.4 Electric Heaters and Thermal Performance

**Figure 4:**
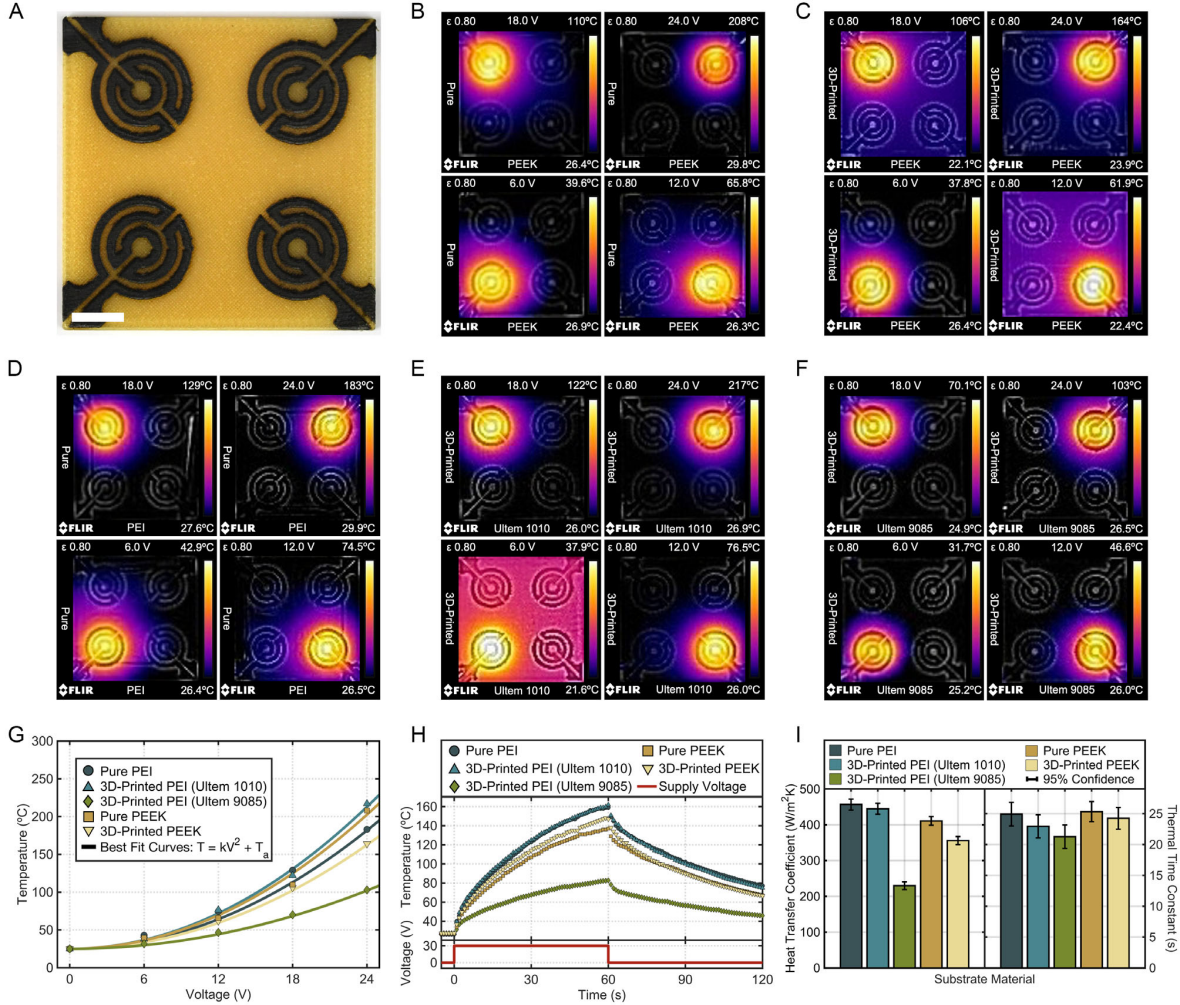
A) LIG serpentine heaters scribed on 3D‐printed Ultem 1010; scale bar 10 mm. Blended IR–visible images of LIG heaters at four different voltages after 200 s on B) pure PEEK, C) 3D‐printed PEEK, D) pure PEI, E) 3D‐printed Ultem 1010, and F) 3D‐printed Ultem 9085. G) Heater temperatures after 200 s as a function of voltage; best‐fit curves are matched to each material's data. H) Heater temperatures as a function of time with a 30 V square wave voltage supply. I) Thermal coefficients of heaters for each precursor's LIG.

Serpentine heating patterns were laser‐scribed on all five precursors to demonstrate the functional heating of LIG circuits and complementary thermal tolerances of PEI and PEEK (**Figure** **4**A). PEI and PEEK possess excellent coefficients of thermal expansion, low thermal conductivities, and some of the highest melting, glass transition, and heat deflection temperatures among all polymers. LIG traces scribed on these polymers responded strongly to voltage differentials via Joule heating, where the electrical current was resistively converted to thermal energy.^[^ ^2^, ^3^, ^4^ ^]^ Beneficially, a conductor's Joule heating is inversely proportional to its sheet resistance (Equation (S1), Supporting Information), so the lowest LIG sheet resistances reported in this study also produced some of the best heater efficiencies. By this equation, thermal flux is also determined by the heater's supply voltage and its geometric aspect ratio; LIG patterns and voltages were chosen so that the heaters did not exceed their precursor's typical operating temperatures. Each substrate's four serpentine heaters were then connected to a DC voltage (6–24 V) and measured with a thermal imaging camera after 200 s of heating (Figure 4B–F).

LIG heaters produced impressive surface temperatures for such high aspect ratios (≈77) and moderate supply voltages: LIG on Ultem 1010 achieved a maximum of 217 °C, pure PEEK with 208 °C, pure PEI with 183 °C, 3D‐printed PEEK with 164 °C, and Ultem 9085 with 103 °C. Moreover, the relation between voltage and temperature matched expected parabolic curves (Equation (S1), Supporting Information) with correlations greater than *R* ^2^ ≥ 0.98 (Figure 4G), providing a strong predictive model to achieve target temperatures with input voltages. Separately, heating distributions along the PEEK surfaces (Figure 4B–C), caused by thermal conduction at the LIG–polymer interface, displayed larger diffusion rings than PEI substrates (Figure 4D–F). This matched the relative strength of thermal conductivity between these materials. However, both PEI and PEEK still showcased high thermal resistance, apparent in their sharp temperature drops away from the center of these diffusion rings.

The time evolution of heaters was also analyzed by measuring temperature changes from a 30 V square wave supply (Figure 4H). All polymers’ LIG exhibited exponential heating for 60 s of supplied voltage, followed by smaller exponential decay for 60 s of removed voltage (Video S1, Supporting Information). A thermodynamic model of this system was derived to characterize the time‐dependency of LIG temperatures (Equation (S1)–(S4), Supporting Information). By this model (Equation (5)), the time‐dependent temperature *T(t)* of the LIG was related to its initial temperature *T*_*i*_, initial temperature flux ${\dot{T}}_{i}$, ambient air temperature *T*_a_, thermal time constant *τ*, surface power density *P*_d_, and heat‐transfer coefficient *h*.

(5) $$T\left(t\right)=\left(1-e^{-t/\tau }\right)\left(\frac{P_{d}}{h}+T_{a}+\tau {\dot{T}}_{i}\right)+T_{i}e^{-t/\tau }$$

This model matched observed data (Figure 4H) with a high degree of correlation on all samples (*R* ^2^ ≥ 0.97). The heat‐transfer coefficients between LIG and ambient air closely matched the order of lowest sheet resistances, with a close grouping between LIG on pure PEI at 457 W m^−2^ K^−1^, Ultem 1010 at 445 W m^−2^ K^−1^, pure PEEK at 411 W m^−2^ K^−1^, 3D‐printed PEEK at 336 W m^−2^ K^−1^, and finally a much lower Ultem 9085 at 230 W m^−2^ K^−1^ (Figure 4I). The surface power densities of each heater were directly related to their LIG's sheet resistance (Equation (S6), Supporting Information), with pure PEI achieving a maximum of 6.62 W cm^−2^. However, the thermal time constants only relied on parameters specific to intrinsic LIG properties, implying that they would not change for other scribed geometries or supply voltages (Equation (S6), Supporting Information). These time constants remained closely between 21 and 25 s for all LIG heaters (Figure 4I), demonstrating that they would achieve 63.2% of their maximum temperature at this time.

### 2.5 Strain Sensors and Mechanical Response

**Figure 5:**
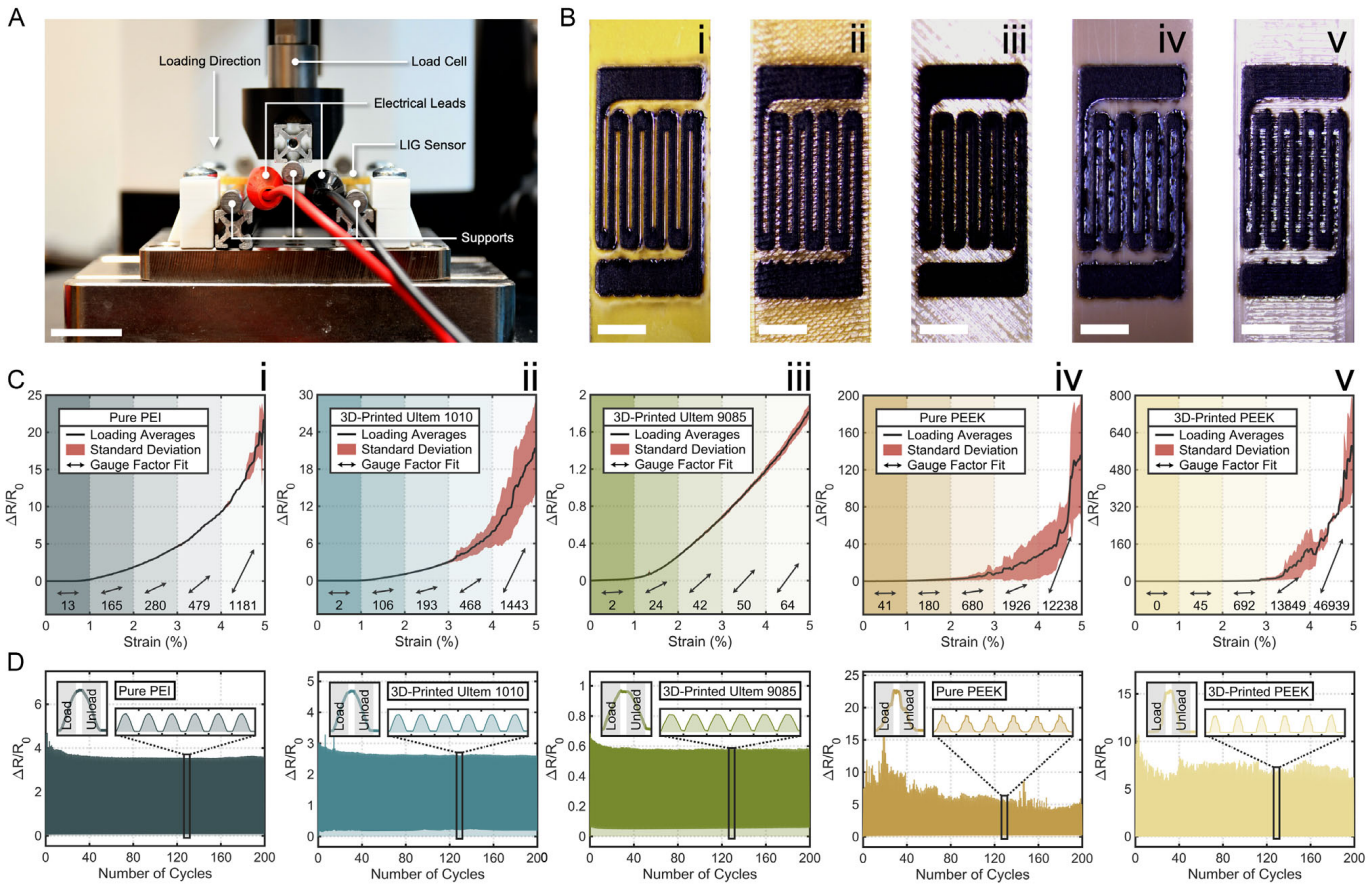
A) Annotated image of three‐point bending setup to measure strain responses of LIG sensors; scale bar 20 mm. B) Images of LIG strain gauges scribed on each material; scale bars 5 mm. C) Average and standard deviations of resistance changes for five samples on each material over 5% flexural strain; gauge factors are provided under associated arrows for every 1% strain increment. All LIG precursors are ordered as i) pure PEI; ii) 3D‐printed Ultem 1010; iii) 3D‐printed Ultem 9085; iv) pure PEEK; and v) 3D‐printed PEEK. D) Strain gauge drifts over 200 flexural cycles up to 3% strain; cyclical strain curves are magnified and labeled for loading and unloading periods.

Excluding fiber‐reinforced composites, PEI and PEEK filaments offer the highest tensile strengths of any FDM thermoplastic. Both materials spearhead the growth of 3D printing in industrial and structural applications, supplemented by high strength‐to‐weight ratios, excellent dimensional stability, and strong resistance to fatigue, creep, impact, and wear.^[^ ^32^, ^33^, ^34^ ^]^ Implementing facile and sensitive strain gauges on these AM components will expand their versatility in real‐time structural monitoring and customizable sensors. To characterize the response of LIG to physical change, uniaxial strain sensors were patterned on flexural specimens from all five PEI and PEEK polymers. Fabricated with the highest studied laser fluence (Φ_0 _= 240 J cm^−2^), these sensors exhibited high conductivities (Figure 3E). Flexural tests were then performed on all strain gauges using a three‐point bending setup (**Figure** **5**A), such that the resistance of LIG sensors increased from elongation (Video S2, Supporting Information). Strain *ε* was calculated at the surface of each specimen from geometric and displacement values; LIG resistance change Δ*R*/*R*_0_ was measured by an inductance (L), capacitance (C), and resistance (R) (LCR) meter.

Despite being fabricated with the same laser parameters, differences in the LIG's patterning resolution on PEI versus PEEK affected subsequent sensing capabilities. Strain gauges scribed on all PEI polymers displayed flat LIG surfaces and sharp edges at their ablated boundaries; however, LIG patterns on PEEK polymers did not (Figure 5B). In extreme cases, pyrolytic jetting dominated the PEEK surface, caused by rapid gas generation and delamination of LIG from the substrate during fabrication^[^ ^5^ ^]^ (Figure S6, Supporting Information). These specimens were not included in flexural tests, but traces of small LIG fibers were observed on all PEEK strain gauges.

Five specimens from each LIG precursor were loaded to 5% flexural strain to compare their resistive responses. Resistance statistics were calculated at each 300 με, and differential gauge factors were recorded for each 1% strain increment (Figure 5C). The maximum gauge factors on each material were substantial: 46 939 for 3D‐printed PEEK, 12 238 for pure PEEK, 1443 for Ultem 1010, 1181 for pure PEI, and 64 for Ultem 1010. Though LIG has demonstrated high (10–100) gauge factors on other polymers,^[^ ^5^, ^6^, ^7^ ^]^ the rationale for these extreme values may be explained by the higher internal surface areas of LIG from PEI and PEEK than other polymers,^[^ ^19^ ^]^ increasing their sensitivity to change.

Although total resistance changes and gauge factors differed between polymers, each material's LIG exhibited internally consistent results. On pure and 3D‐printed PEI, the coefficient of variation (CV) between individual strain gauges was less than 0.061 for strains less than 3%, indicating high reliability between samples (Figure S7, Supporting Information). LIG on 3D‐printed Ultem 9085 performed particularly well (CV ≤ 0.032) with highly linear responses (*R* ^2 ^≈ 0.99) from 1.5% to 5% strain. This performance can be attributed to the interwoven network on Ultem 9085's LIG (Figure 2B,C), which is likely more flexible than the stiffer sheets of other LIG samples. LIG has been shown to produce prominent gauge factors from shifting its graphene layers, which changes their contact resistance and diminishes the number of available electrical pathways.^[^ ^5^ ^]^ However, lattice structures can endure much higher strains than solid formations without rupture. This explains why Ultem 9085's LIG sensors were simultaneously more linear, consistent, and lower in gauge factor than others.

Conversely, pure and 3D‐printed PEEK put forth highly nonlinear signals, significant inconsistencies between samples (CV ≤ 0.92), and immense gauge factors (Figure 5C). This erratic behavior was repeated when flexural specimens were cyclically loaded to *ε* = 3% over 200 cycles (Figure 5D). These signals from PEEK samples were likely caused by residual LIG fibers formed by pyrolytic jetting. Like the shifting of layered graphene within LIG, loose LIG fibers may have shifted on the PEEK surface during flexural loading. Considering the relative size of these fibers (≤200 μm, Figure S6, Supporting Information) to LIG sheets (≤1 μm, Figure 2C), discrete resistive jumps were more apparent from larger and fewer electrical discontinuities. Accordingly, LIG on PEEK specimens exhibited more inconsistent signals than non‐fibrous PEI samples across individual (Figure 5D) and multiple (Figure 5C) samples.

Over the 200 loading cycles, pure PEI, Ultem 1010, and Ultem 9085 produced highly stable responses after ≈20 cycles with minimal drift (CV ≤ 0.013). Noticeably, all materials exhibited settling behaviors, where stable Δ*R*/*R*_0_ numbers dropped by ≈83% from their initial values at *ε* = 3%. However, considering the minimal creep of both PEI and PEEK polymers, this change may be due to permanent microcracking of the LIG.^[^ ^6^, ^7^ ^]^ After this settling, the uniformities of entire loading curves were apparent when overlaid on gauge factor plots (Figure S8, Supporting Information). Cyclical paths displayed little deviation over the 200 cycles; however, loading paths followed distinctly separate routes than unloading curves. The emergence of this hysteresis may have been amplified by a pause at *ε* = 3% between loading and unloading, during which stress relaxation of LIG caused the sensor's resistance to drop.

## 3 Conclusion

LIG electronics were fabricated on PEI and PEEK from pure and 3D‐printed specimens with a single‐step laser scribing process (*λ* = 450 nm, *P*_L_ = 0.1–1.5 W). Stable minimum sheet resistances of LIG (1.02 to 3.79 Ω sq^−1^) were achieved across a range of process parameters. The high conductivity of this LIG (≤45.4 S cm^−1^) was attributed to reduced porosity in its microstructure, high carbonization in its interior, and abundant nitrogen doping in its atomic composition. Though the high concentration of this nitrogen (≤16.3%) was ascribed to the ionization of ambient N_2_ molecules during LIG formation, the exact mechanism of how the formative blue laser facilitated these covalent C—N bonds needs to be studied further. The sheet resistance of each LIG sample was also found to be strongly correlated to its formative laser fluence (*R* ^2^ ≤ 0.94), and a new empirical model was presented relating the two parameters. Joule heaters and strain gauges were laser‐scribed on pure and 3D‐printed samples to demonstrate the versatility of LIG electronics. PEI and PEEK tolerate high thermal and mechanical stresses, making them ideal precursors for these applications. LIG heaters operated up to the limits of each polymer's glass transition temperature with large heat‐transfer coefficients (≤ 457 W m^−2^ K^−1^) and low thermal time constants (≈25 s). LIG strain sensors on pure and 3D‐printed PEI displayed high stability (CV ≤ 0.013) up to 3% strain and over 200 loading cycles. Furthermore, 3D‐printed Ultem 9085 exhibited high linearity (*R* ^2^ ≤ 0.99) from 1.5% to 5% strain with slight variations between specimens (CV ≤ 0.032). These findings demonstrate that conductive, heating, and sensing LIG electronics can be laser‐scribed directly on high‐performance 3D‐printed structures with low laser powers (≤1.5 W). Future AM with integrated laser systems will allow the fabrication of customizable 3D components with embedded LIG electronics for multifunctional applications.

## 4 Experimental Section

4.1

4.1.1

##### Preparation of Precursor Specimens

Ultem 1010 resin filaments (Stratasys, Eden Prairie, MN) and Ultem 9085 resin filaments (Stratasys) were FDM printed by a Fortus 900mc printing system (Stratasys) with a build volume of 914 by 610 by 914 mm. ThermaX PEEK filament (3DXTech, Grand Rapids, MI) were FDM printed by a 22 IDEX printer (Vision Miner, Santa Ana, CA) with a build volume of 350 by 350 by 450 mm. All filaments had an initial diameter of ø1.75 mm and were printed with rectilinear solid infills and layer heights of 0.33 mm. Sheets of pure Ultem PEI (Cat. No. 8685K43) and PEEK (Cat. No. 8504K21) were purchased from McMaster‐Carr (Elmhurst, IL) with a thickness of 1.59 mm, which matched the build heights of all 3D‐printed samples. Rectangular substrates prepared for SEM and EDS tests had dimensions of 5 mm; those for Raman spectroscopy, electrical, and heating tests had dimensions of 60 mm; and those for flexural tests had lengths and widths of 50.8 by 12.7 mm following ASTM D790‐17. Pure polymers were cut by a bandsaw and smoothed on a grinder to these specifications with tolerances of ±0.1 mm. No abrasive surface treatment was conducted on any area that would be laser‐scribed for LIG.

##### Synthesis of LIG Patterns

Specimens were cleansed with isopropyl alcohol, left to dry in ambient conditions for ≈60 s, then loaded on the build platform of a Snapmaker Original 3‐in‐1 3D printer (Snapmaker, Shenzhen, China). Equipped with a 1.6 W blue laser diode (*λ* = 450 nm), this system ablated the surface of each sample according to the parameters presented in Section 2.1. Scribed patterns and corresponding G‐code were either designed in Inkscape and emitted from Snapmaker Luban software or both designed and emitted from custom MATLAB code. After scribing, LIG samples were kept in a dust‐free environment until further testing.

##### Morphological and Spectroscopy Characterization

SEM images and EDS data were taken with a Nova NanoSEM 450 field‐emission SEM (FEI Company, Hillsboro, OR) with magnifications of 650–3500×, charging voltages between 1 and 10 keV, and beam spot sizes of 3–4 nm. Side‐view SEM images were procured by irradiating LIG over the edge of its precursor and scraping away excess graphitization with a razor blade, revealing the LIG–polymer interface. LIG thicknesses were measured on these substrates using low‐magnification SEM images (Figure S1, Supporting Information); averages and standard deviations were calculated from 10 equally spaced LIG heights across the images. Raman spectra were obtained using an inVia Raman Microscope (Renishaw, Wotton‐under‐Edge, UK) with a laser excitation wavelength of 532 nm and power of 3–5 mW. Baseline subtraction, peak normalization, and intensity ratio processing of the spectra were conducted using custom MATLAB code.

##### Electrical Characterization

Sheet resistances were measured with an SP4 Four‐Point Probe Head (Signatone, Gilroy, CA) and a 34401A Digital Multimeter (Hewlett‐Packard Company, Palo Alto, CA), which together provided a sampling precision of 0.001 Ω sq^−1^. The equipment was first checked for calibration against a silicon wafer doped with a 50 nm layer of copper, which matched its target sheet resistance at 0.150 Ω sq^−1^ with an error of 0.005 Ω sq^−1^. Two measurements were then conducted on each of the 320 LIG samples for averages and standard deviations.

##### Thermal Testing

A circular serpentine heating pattern was scribed on all LIG precursors with a 1.5 mm path width, 1.5 mm path spacing, and 116 mm path length (Figure S9, Supporting Information). Surface temperatures of LIG heaters were measured by an FLIR E5 Infrared Camera (Teledyne Technologies, Thousand Oaks, CA) with a 120 by 90 pixel resolution, −20 to 250 °C temperature range, and 0.10 °C thermal sensitivity. Maximum temperatures were measured ≈100 mm away from the heaters’ surfaces to increase imaging resolution, while thermal multi‐spectral dynamic imaging (MSX) images were taken afterward at ≈300 mm to reduce parallax between thermal and visual images.

##### Flexural Testing

Uniaxial strain gauge patterns were scribed on flexural samples with nine 0.5 mm loops spaced 0.75 mm apart for a total gauge width and length of 10.5 mm (Figure S9, Supporting Information). Specimens were then placed in a three‐point bending setup (32.0 mm span, ø6 mm support pins) while an LCR‐6100 Precision LCR Meter (GW Instek, Taipei, Taiwan) measured strain gauge resistance. The loading nose was attached to an M7‐200 Force Gauge (Mark‐10, Copiague, NY) to measure force and displacement, which was positioned by an ESM303 Motorized Test Stand (Mark‐10). The LCR meter was synchronized to positional measurements, and both provided data at 8 Hz. Initial strain (*ε* = 0) was calculated from the displacement at which the force probe first detected the flexural resistance of the specimens. Loading rates were conducted at 15 mm min^−1^ for single flexural tests up to 5% strain; rates were increased to 60 mm min^−1^ during cyclical loading to 3% strain.

## Conflict of Interest

The authors declare no conflict of interest.

## Author Contributions

**Joshua Vandervelde**: conceptualization (lead); data curation (lead); formal analysis (lead); investigation (equal); methodology (lead); project administration (lead); software (lead); validation (equal); visualization (lead); writing—original draft (lead); and writing—review and editing (equal). **Yeowon Yoon**: investigation (equal); validation (equal); and writing—review and editing (equal). **Rifat Shahriar**: investigation (supporting) and resources (equal). **Steven B. Cronin**: resources (equal) and writing—review and editing (supporting). **Yong Chen**: conceptualization (supporting); funding acquisition (lead); resources (equal); supervision (lead); and writing—review and editing (equal).

## Supporting information

Supplementary Material

## Data Availability

The data that support the findings of this study are available in the supplementary material of this article.
