# Supplementary material for: Additive and Laser Manufacturing for Multifunctional Electronics on High‐Performance Polymers
Source: Small Sci. 2025 Apr 2;5(6):2500022. doi: 10.1002/smsc.202500022 (PMC12168607; doi:10.1002/smsc.202500022)
Supplement: Supplementary file 1 — Supplementary Material [file SMSC-5-2500022-s001.zip › smsc12703-sup-0001-suppdata-S3.pdf]

Additive and Laser Manufacturing for Multifunctional Electronics on High-Performance Polymers

*Joshua Vandervelde, Yeowon Yoon, Rifat Shahriar, Stephen B. Cronin, and Yong Chen**

J. Vandervelde, Y. Yoon, Y. Chen
Center for Advanced Manufacturing, University of Southern California, Los Angeles, CA 90007, USA
Department of Aerospace and Mechanical Engineering, University of Southern California, Los Angeles, CA 90089, USA
*Email: yongchen@usc.edu

R. Shahriar, S. B. Cronin
Department of Electrical Engineering, University of Southern California, Los Angeles, CA, 90089, USA

S. B. Cronin
Department of Physics and Astronomy, University of Southern California, Los Angeles, CA, 90089, USA
Department of Chemistry, University of Southern California, Los Angeles, CA, 90089, USA

Y. Chen
Department of Industrial and Systems Engineering, University of Southern California, Los Angeles, CA 90089, USA
Department of Biomedical Engineering, University of Southern California, Los Angeles, CA 90089, USA

Supporting Information

Table S1. A summary of formative laser parameters and precursor materials that influence the lowest sheet resistances of LIG reported in the literature.

Reference Index	Laser Power (W)	Laser Wavelength (nm)	Precursor Material	Sheet Resistance (Ω/sq)
[1]	5.4	10600	PI	15
[3]	0.1	405	PI	250
[5]	0.5	405	PI	80
[5]	0.8	343	Organic Material	10
[5]	0.8	1064	Organic Material	40
[5]	8.6	10600	Organic Material	10
[5]	9	10600	PI	100
[7]	4	10600	PEEK	120
[9]	6	10600	PI	113
[10]	2.8	10600	Organic Material	11

[10]	2.8	10600	Organic Material	46
[10]	2.8	10600	Organic Material	55
[15]	3	405	PET	90
[22]	0.16	405	PI	108
[25]	5.5	450	Organic Material	345
[26]	2.5	450	PC	500
[27]	0.6	532	PI	6.04
[27]	0.8	532	PEI	3.62
[29]	3	10600	PEI	186
[29]	7	10600	PI	19.8
[35]	4.5	10600	PEI	16
[42]	12	10600	PEI	0.3
[42]	12	10600	PEI	1.79
[46]	3.75	10600	Organic Material	8
[46]	3.75	10600	PEI	15
[48]	7	10600	PEI	20
[52]	0.46	460	Organic Material	52
[52]	0.7	347	PPTA	5.02
[52]	0.9	450	Organics	46
[52]	1.5	1035	PPTA	2.86
[52]	1.8	450	Organic Material	10.8
[52]	2.7	10600	Organic Material	3.8
[52]	3.6	10600	Organic Material	2.8
[52]	4.5	10600	PPXC	9.2
[52]	5.5	1060	Organic Material	7.5
[52]	6	10600	PES	4.1
[52]	12	10600	Organic Material	4.5
[52]	12	10600	PDMS	130
This Study	0.1	450	PEEK	21.4
This Study	0.3	450	PEEK	2.99

This Study	0.5	450	PEEK	1.63
This Study	0.7	450	PEEK	1.44
This Study	0.9	450	PEEK	1.17
This Study	1.1	450	PEEK	1.12
This Study	1.3	450	PEEK	1.13
This Study	1.5	450	PEEK	1.25
This Study	0.1	450	PEI	16.3
This Study	0.3	450	PEI	2.78
This Study	0.5	450	PEI	1.68
This Study	0.7	450	PEI	1.28
This Study	0.9	450	PEI	1.22
This Study	1.1	450	PEI	1.17
This Study	1.3	450	PEI	1.08
This Study	1.5	450	PEI	1.02

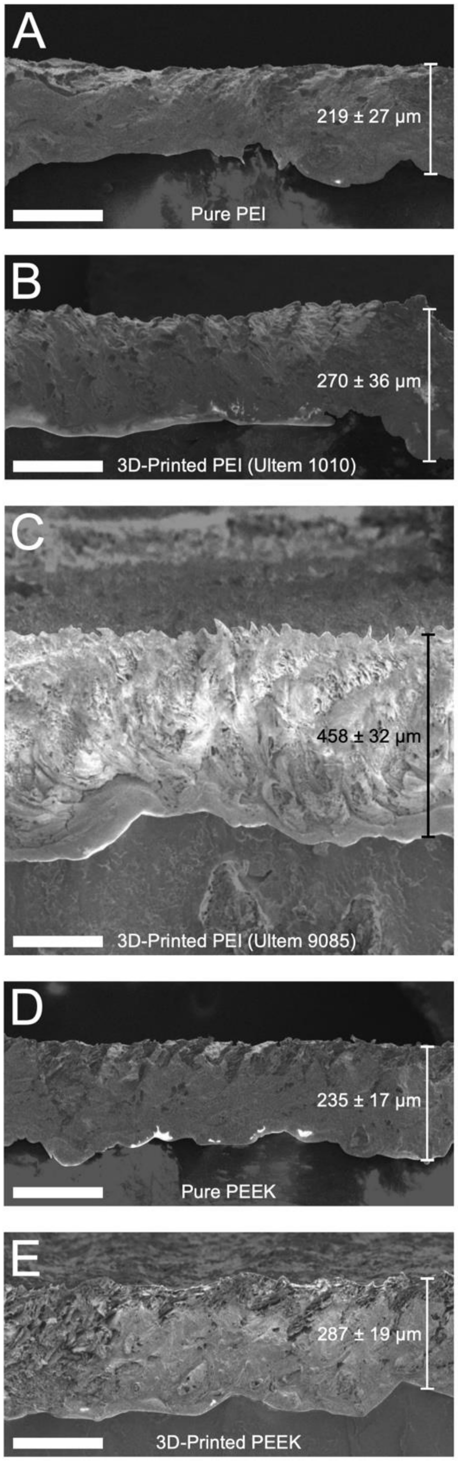

Figure S1. Side-view SEM images are used to measure average LIG thicknesses across the entire imaged width of A) pure PEI, B) 3D-printed Ultem 1010, C) 3D-printed Ultem 9085, D) pure PEEK, and E) 3D-printed PEEK. All scale bars are 200 µm.

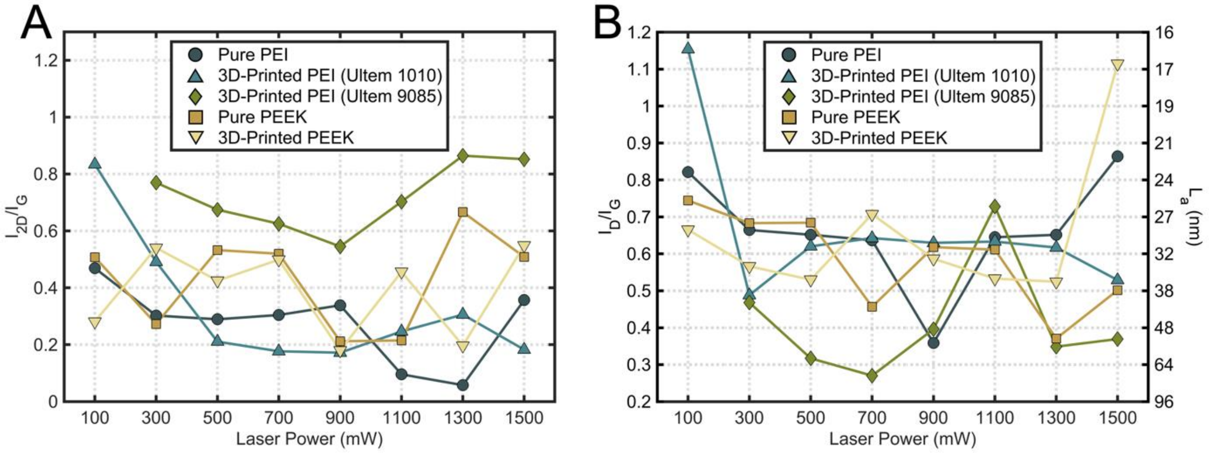

Figure S2. A) Intensity ratios of 2D and G peaks (I_{2D}/I_G) from Raman spectra of LIG over different laser powers. B) Intensity ratios of the D and G peaks (I_D/I_G) with associated crystalline lengths of the graphene.

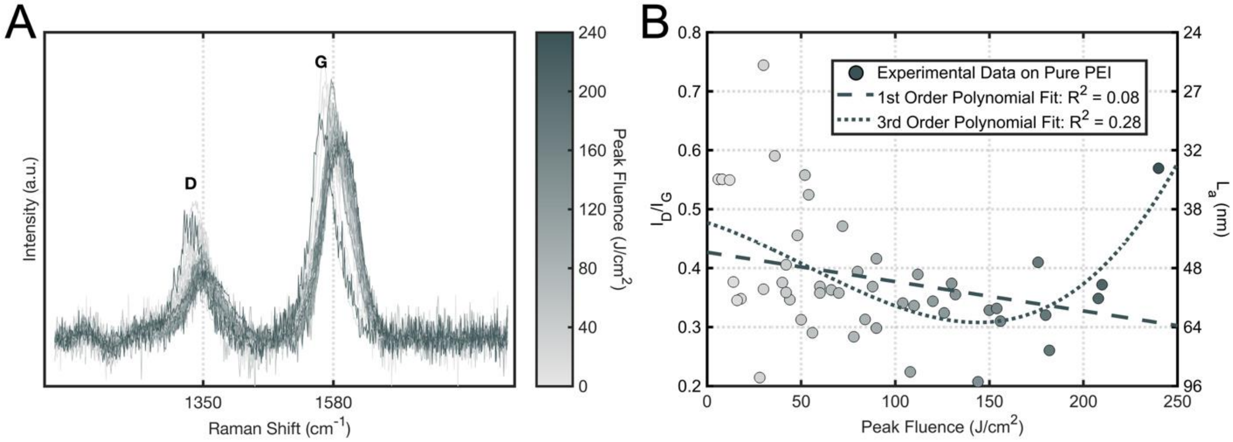

Figure S3. A) Raman spectra of LIG on pure PEI from all combinations of laser power and pulse durations. B) Low correlations between laser fluence and I_D/I_G values from first- and third-order polynomial best-fit curves.

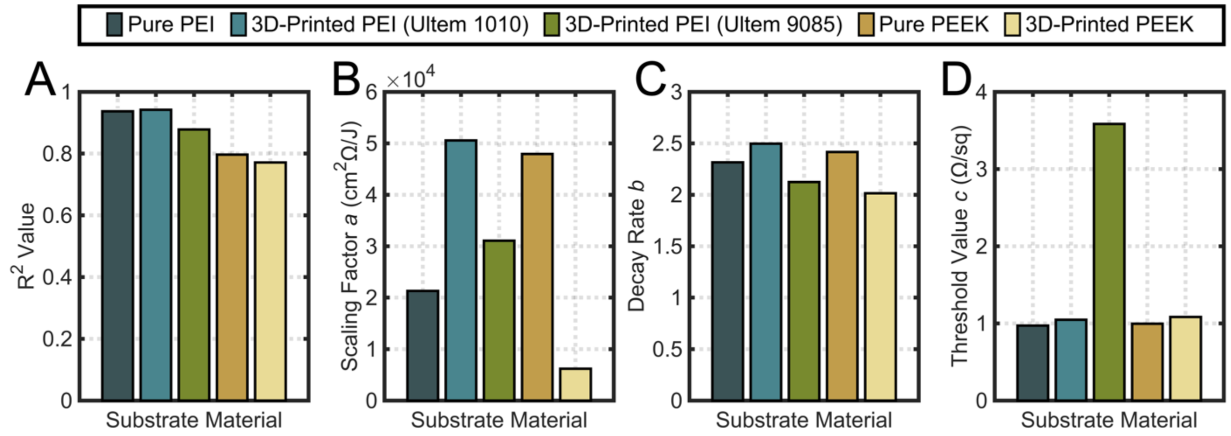

Figure S4. Coefficient values of best-fit curves between polymers' LIG sheet resistance and laser fluence for A) determination coefficients, B) scaling factors, C) decay rates, and D) threshold values.

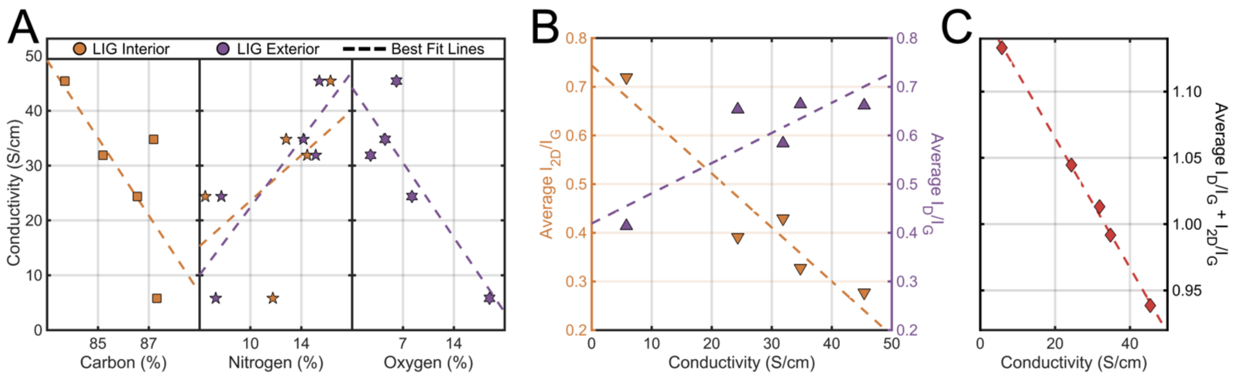

Figure S5. A) Changes in all precursors' LIG conductivities with respect to concentrations of interior carbon (R^2 ~ 0.54), interior nitrogen (R^2 ~ 0.27), exterior nitrogen (R^2 ~ 0.74), and exterior oxygen (R^2 ~ 0.72) from EDS measurements. B) Effects on LIG conductivity on average Raman spectra I_{2D}/I_G ratios (R^2 ~ 0.90) and I_D/I_G ratios (R^2 ~ 0.73). C) Correlation between LIG conductivity and composite Raman $I_D/I_G + I_{2D}/I_G$ ratio (R^2 ~ 0.99).

Discussion on Heater Temperature Model

The surface temperature on LIG heaters was found using the first law of thermodynamics. The examined system encompassed the LIG pattern, while the system's environment included both the LIG's substrate and surrounding air. Due to the excellent thermal resistivities of PEI and PEEK and minimal radiation of LIG at these temperatures, heat losses from conduction and radiation were assumed to be negligible compared to convection. Since the heaters were also assumed to exhibit isotropic heating along their serpentine paths ($\partial Q/\partial A \sim 0$), heat flux was dominated by the heat energy's temporal change. By this convention, the heat flux generated by Joule heating (Equation S1) and the heat flux of convective losses to ambient air (Equation S2) determined the internal heat flux of the system (Equation S3).

$$\dot{Q}_{Joule} = \frac{V^2}{R_{sh}\,L/W} \tag{1}$$

$$\dot{Q}_{Convection} = hLW(T(t) - T_a) \tag{2}$$

$$\dot{Q}_{LIG} = mc_p\big(\dot{T}(t) - \dot{T}_i\big) \tag{3}$$

Equating these three terms produced the time derivative of the conservation of heat energy (Equation S4). Though the air surrounding the system increased in thermal energy and would have affected both the heat transfer rate from LIG to air and the total ambient temperature, these changes were assumed to be small.

$$\dot{Q}_{LIG} = \dot{Q}_{Joule} - \dot{Q}_{Convection} \tag{4}$$

After solving this first-order linear ordinary differential equation for the time evolution of the system's temperature (Equation 5), terms could be repackaged into meaningful thermal coefficients. The heater's surface power density (Equation S5) scaled linearly against the inverse of its sheet resistance, in the same manner as Joule heating, but increased by the square of its voltage and inverse electrical path length. This implied that heater geometry had a more pronounced effect on the maximum heater temperature than on Joule heat energy.

$$P_d = \frac{1}{R_{sh}}\left(\frac{V}{L}\right)^2 \tag{5}$$

The thermal time constant (Equation S6) of the LIG was also found with mass *m* and surface area terms *L* and *W* replaced by density ρ and thickness *d*. This rearrangement highlighted that τ remained the same for LIG with a given specific heat capacity c_p and heat transfer coefficient to air *h*, irrespective of geometric or supply voltage changes.

$$\tau = \frac{\rho d c_p}{h} \tag{6}$$

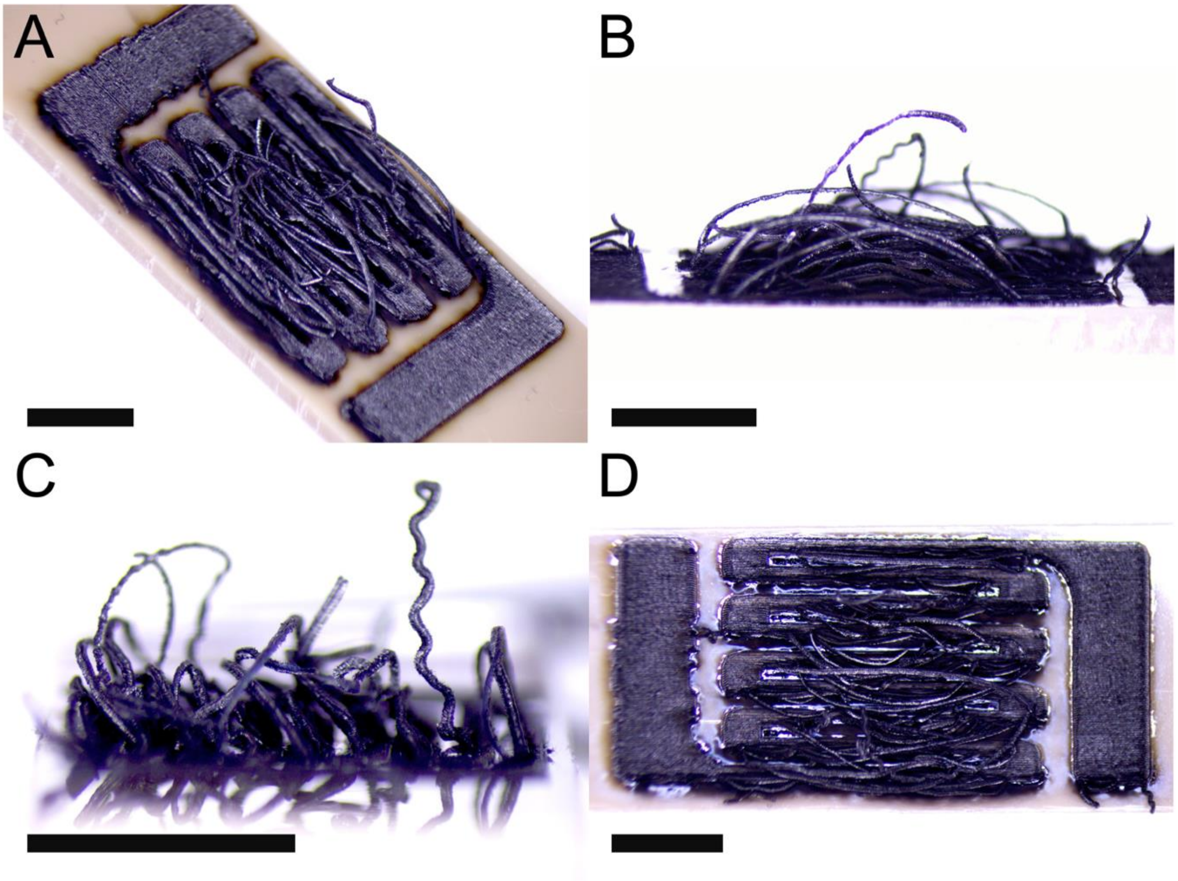

Figure S6. Pyrolytic jetting on a LIG strain gauge from a PEEK specimen, seen from A) isometric view, B) side view, C) front view, and D) top view. All scale bars are 5 mm.

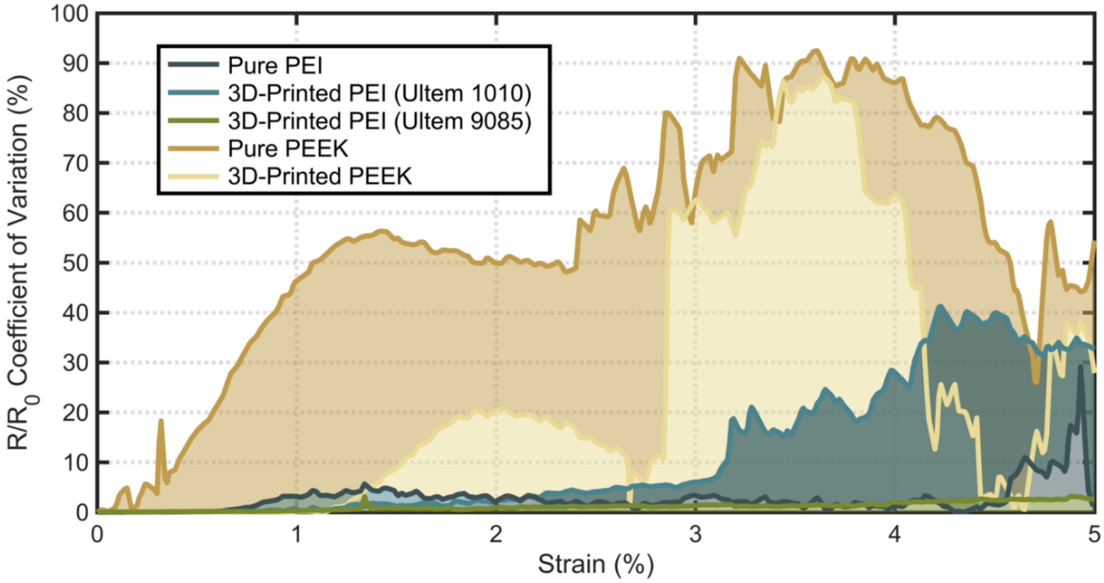

Figure S7. Coefficients of variation between normalized resistances between multiple LIG strain gauge samples from each polymer.

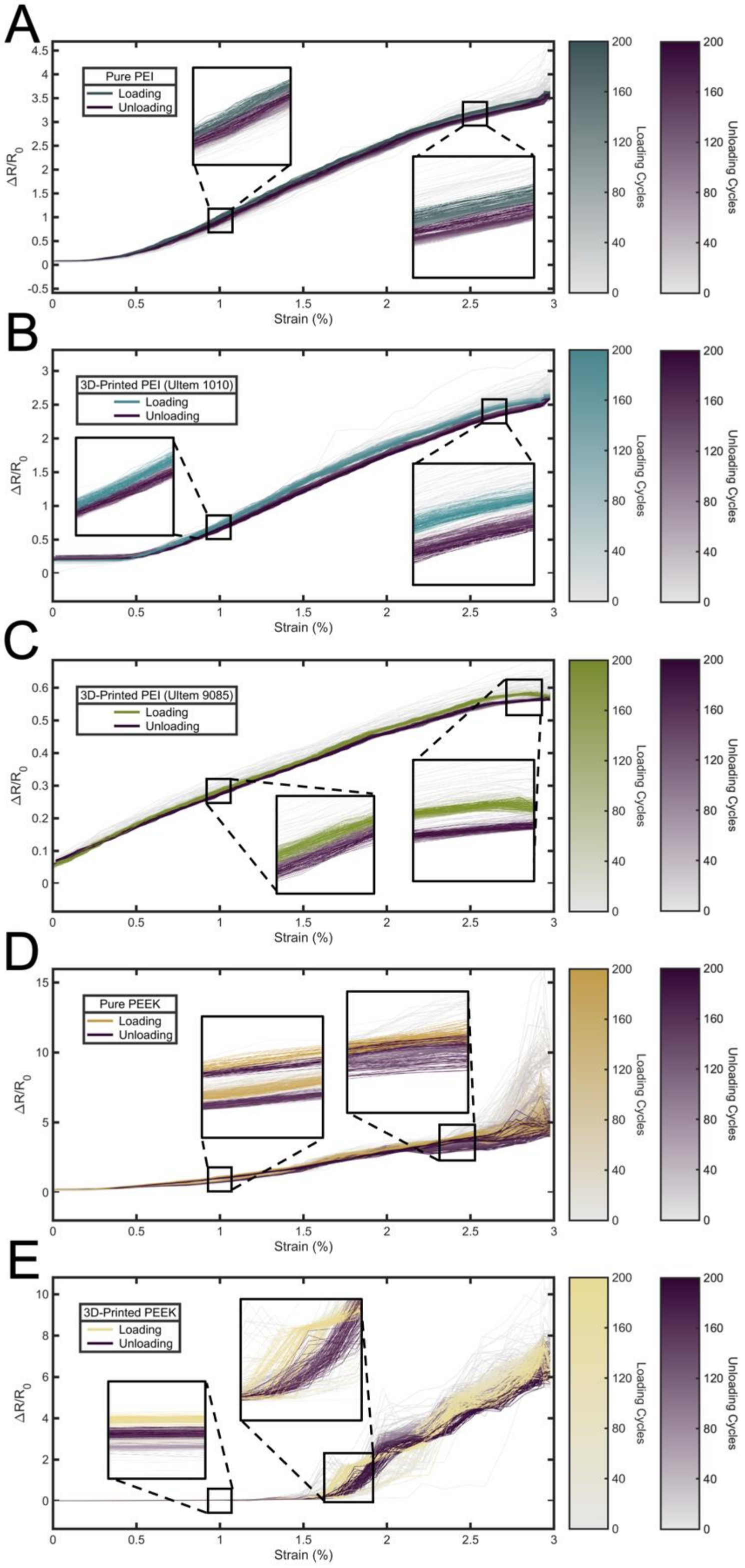

Figure S8. Stress cycles and strain settling on LIG samples from A) pure PEI, B) 3D-printed Ultem 1010, C) 3D-printed Ultem 9085, D) pure PEEK, and E) 3D-printed PEEK. Magnified sections display hysteresis between loading and unloading resistance changes.

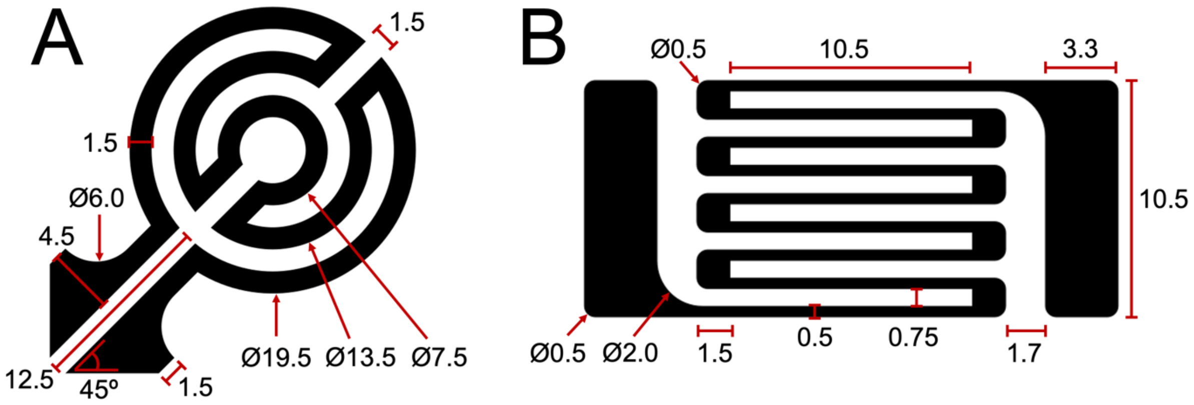

Figure S9. A) Dimensions of circular serpentine heaters for functional LIG electronics; all units in mm. B) Dimensions of uniaxial strain gauges for sensing LIG electronics; all units in mm.
